# Intravenous administration of activated protein C in *Pseudomonas*-induced lung injury: impact on lung fluid balance and the inflammatory response

**DOI:** 10.1186/1465-9921-7-41

**Published:** 2006-03-22

**Authors:** Laurent Robriquet, François Collet, Antoine Tournoys, Thierry Prangère, Rémi Nevière, François Fourrier, Benoît P Guery

**Affiliations:** 1 EA 2689, Faculté de Médecine–Université de Lille 2- 1 place de Verdun, 59045 Lille Cedex, France; 2 Laboratoire d'Hématologie CHRU Lille, Hopital Salengro, Bd Pr Leclecq, 59037 Lille Cedex, France; 3 Laboratoire de Biophysique- Service de Médecine Nucléaire–Faculté de Médecine/CHRU de Lille- 1 place de Verdun, 59045 Lille Cedex, France

## Abstract

**Background:**

Acute lung injury (ALI) induces a coagulation/fibrinolysis imbalance and leads to fibrin deposition. The protein C pathway is an important regulator of the coagulation system and reduces the inflammatory response. The aim of the study was to examine the effects of recombinant human activated protein C (rhAPC) in the early phase of *Pseudomonas aeruginosa *(*Pa*)-induced lung injury.

**Methods:**

The study was conducted in vivo on a rat model of *Pa*-induced ALI. Continuous intravenous (IV) rhAPC was administrated simultaneously with intratracheal (IT) *Pa*. We instilled into the airspaces a 5% bovine albumin solution with 1 μ(Ci of ^125 ^I-albumin and injected IV 1 μ(Ci of ^111^In-albumin to measure lung liquid clearance (LLC) and endothelial injury. Cytokines levels (TNFα and IL-6) and thrombin-antithrombin (TAT) complexes were measured in blood and bronchoalveolar lavage fluid (BALF) at 4 hours. Four groups were compared: control (CTR), pneumonia (PNP) receiving IT *Pa *(0.5 ml/kg of 1 × 10^9 ^cfu), APC: IV rhAPC (300 μg/kg/h), A-PNP: IT *Pa */IV rhAPC.

**Results:**

Alveolar-capillary permeability was increased in the PNP versus the CTR group (0.28 ± 0.08 vs. 0.03 ± 0.01, p < 0.05). IV rhAPC in *Pa*-induced ALI led to further injury (0.47 ± 0.17 vs. 0.28 ± 0.08, p = 0.2). The LLC was significantly decreased in the A-PNP group compared to PNP group (9.1 ± (4.3% vs. 33.4 ± 2.6%, p < 0.05). The lung wet to dry weight ratio was significantly increased in the PNP group (4.62 ± 0.31) compared to the CTR group (3.87 ± 0.22, p < 0.05). IV rhAPC administration tends to increase this parameter in *Pa*-induced ALI (5.80 ± 0.66, p = 0.07). These findings were associated with a loss of inflammatory response compartmentalization measured by TNFα and IL-6 systemic levels. TAT complexes in BALF were increased in the A-PNP group (23.17 ± 2.89 ng/ml) compared to the CTR group (0.92 ± 0.17 ng/ml, p < 0.05) and the PNP group (11.06 ± 2.76 ng/ml, p < 0.05).

**Conclusion:**

rhAPC reduces LLC following *Pa*-induced ALI and may influence pulmonary edema formation. The early massive fibrin formation is probably beneficial in ALI limiting both the extent of injury and permeability disorders.

## Background

*Pseudomonas aeruginosa *(*Pa*) is one of the most frequent pathogens involved in nosocomial pneumonia [1]. This Gram-negative bacillus is associated with a mortality rate reaching 70% in some studies and an attributable mortality approaching 40% [2]. Instillation of this pathogen into the air spaces increases the protein permeability of the lung barrier to induce both interstitial and alveolar edema [3]. Active ion transport is the primary mechanism driving alveolar liquid clearance in the normal lung [4]. The maintenance of active ion transport is critical for the resolution of alveolar edema [5,6]. Moreover, improvement of fluid reabsorption from the air spaces in the *Pa*-induced lung injury in an experimental rat model of pneumonia is associated with a decrease in mortality [7].

Despite the improvement of ventilatory strategies, Acute Respiratory Distress Syndrome (ARDS), the most severe form of acute lung injury (ALI), is associated with a mortality estimated to 35 % [8]. ALI is characterized by a sequence of pathological events which can be subdivided into three phases: the acute or exsudative phase, a sub-acute or proliferative phase, and finally a chronic or fibrotic phase [9,10]. During the exsudative phase, extensive epithelial and endothelial damages result in intra-alveolar deposition of fibrin [11], which is the hallmark of a shift in the coagulation/fibrinolysis balance.

Several studies focused on this pro-coagulative imbalance during ALI: an increase in bronchoalveolar pro-coagulant activity associated with a depressed bronchoalveolar fibrinolysis activity has been found in animal models as well as humans [12-14]. In fact, depressed bronchoalveolar urokinase plasminogen activator activity was found in clinical ARDS [15], this decreased bronchoalveolar fibrinolysis activity was associated with an increased fibrinolysis inhibition related to plasminogen activator inhibitor (PAI-1) [14]. As a consequence to this procoagulant activity associated with decreased fibrinolysis activity, a fibrotic evolution could be triggered [14,16] and influence patient's outcome [17]. From these data, the modulation of the lung coagulation/fibrinolysis balance appears to be a potential therapeutic approach in ALI and ARDS [18].

Protein C is a natural anticoagulant with an important role in coagulation homeostasis through proteolytic cleavage and inactivation of factors Va and VIIIa [19]. Activated protein C has also been shown to restore a pro-fibrinolytic activity by inhibiting PAI-1, and to reduce the inflammatory response through a limitation of thrombin generation and a modulation of nuclear factor-(B translocation [19]. In vivo, activated protein C reduces pulmonary injury in a model of LPS-induced ALI [20]. Moreover, a therapeutic effect was observed in septic baboons [21] and in humans severe sepsis with a major decrease in mortality [22].

The aim of our study was therefore to evaluate, in a rat model, the effects of recombinant human activated protein C (rhAPC) in the early phase of *Pseudomonas aeruginosa*-induced lung injury. We evaluated the effect of rhAPC co-administration in a rat model of *Pa*-induced lung injury on lung permeability and lung fluid movement. Finally, alveolar and systemic cytokines were measured along with coagulation parameters to complete these functional studies.

## Methods

### 1. Animals

Specific pathogen-free Sprague Dawley rats (n = 50) (280–320 g, Charles River Laboratoires France, St Germain/l'Arbresle, France) were housed in the Lille University Animal Care Facility and allowed food and water ad lib. All experiments were performed with approval of the Lille Institutional Animal Care and Use Committee.

### 2. In vivo study

#### 2.1. Surgical preparation

Sprague-Dawley male rats were anesthetized with pentobarbital (Sanofi, Libourne, France). An endotracheal tube (PE-220) was inserted through a tracheotomy. The rats were ventilated with a constant volume pump (Harvard Apparatus, South Natick, MA), with an inspired O_2 _fraction of 1.0, a respiratory rate of 60/min and a positive end expiratory pressure of 2 cmH_2_O. A catheter (PE-50) was inserted into the left carotid artery to monitor systemic arterial pressure and obtain blood samples. Low molecular weight heparin was injected into the catheter (100 UI/kg). Pancuronium bromide (0.3 mg/kg/h iv) was administered to achieve neuromuscular blockade.

#### 2.2. General protocol

For all experiments, the following general protocol was used. After the surgical preparation, heart rate and blood pressure were allowed to stabilize. The rat was then placed in left lateral decubitus to facilitate liquid deposition into the left lung.

To calculate the flux of plasma protein into the lung interstitium, a vascular tracer, 1 μCi of ^111^In-labeled human serum albumin (HSA), was injected into the bloodstream [7,23]. ^111^In-HSA was prepared in our institution according to a standardized technique [24]. To calculate the alveolar liquid clearance, 3 ml/kg of a 5% bovine albumin solution containing 1 μCi of ^125 ^I-albumin were instilled 30 min later into the left lung over a 2-min period, using a 1-ml syringe and polypropylene tube (0.5 mm internal diameter) [7,23].

One hour after the beginning of albumin instillation, the abdomen was opened, and the rat was exsanguinated. Urine was sampled for radioactivity levels. The lungs were removed through a sternotomy, and fluid from the distal airspaces was obtained by passing a propylene tube (0.5 mm internal diameter) into a wedged position in the left lower lobe. The total protein concentration and the radioactivity of the liquid sampled were measured. Right and left lungs were homogenized separately for water to dry weight ratio measurements and radioactivity levels.

#### 2.3. Preparation of albumin instillate

The test solution used for albumin instillation was prepared as follows: briefly, a 5% bovine albumin solution was prepared using Ringer lactate and adjusted with NaCl to be isoosmolar with the rat circulating plasma [7,23]. We added 1 μCi of ^125 ^I-labeled human serum albumin (^125 ^I-HSA; CIS biointernational, Gif sur Yvette, France) to the 5% albumin solution. Anhydrous Evan's blue dye (0.5 mg) was added to confirm the location of the instillate at the end of the study. A sample of the instilled solution was saved for total protein measurement, radioactivity counts, and water to dry weight ratio measurements so that the dry weight of the protein solution could be substracted from the final lung water calculation.

#### 2.4. Measurements

##### Hemodynamics, pulmonary gas exchange, and protein concentration

Systemic arterial pressure and airway pressures were measured continuously (Acknowledge Software v3.7.1, Biopac Systems, Santa Barbara, CA, USA). Arterial blood gases were measured at one-hour intervals (ABL 520, Radiometer, Copenhagen). The arterial PO_2 _was used to quantify the oxygenation deficit [25]. Samples from instilled protein solution, final distal airspace fluid, initial and final blood were collected to measure total protein concentration with an automated analyzer (Hitachi 917, Japan).

##### Lung barrier function study

Permeability of the alveolar-capillary barrier was assessed with the measure of the albumin flux. ^111^In-albumin counts in the final airspace sample were compared to plasma ^111^In-albumin counts averaged over the course of the experiment; permeability to the tracer was expressed as a ratio of airspace to plasma counts (Asp/Plasma). As previously shown [3], this ratio provides a good index of equilibration of the vascular protein tracer into the alveolar compartment.

Accumulation of the vascular protein tracer into the extravascular space of the lung was calculated by determining the total extravascular count of ^111^In-albumin in the lung divided by the average counts in the plasma over the study period and was expressed as total plasma equivalents (TPE) in microliters [26].

##### Lung liquid clearance

The extravascular lung water (EVLW) was measured by the gravimetric method [7,25]. For each animal, the noninstilled lung was used to calculate EVLW and estimate lung liquid clearance (LLC) [25,25].

##### Alveolar fluid movement

Changes of native bovine albumin concentration over the study period (1 h) were used to measure alveolar fluid movement. The term "alveolar" does not imply that all fluid reabsorption occurred at the alveolar level, since some reabsorption may have occurred across distal bronchial epithelium.

The alveolar liquid clearance (ALC) was measured by the ratio of the final unlabeled alveolar protein concentration, compared to the initial instilled alveolar protein concentration (ALC_prot_) [23,27]. The concentrations of alveolar ^125 ^I-albumin were also used to estimate alveolar liquid clearance (ALC_125_) [27].

Measuring alveolar liquid clearance with this methodology relies mainly on the calculation of the alveolar volume, which may, in the context of ALI, be falsely evaluated. We therefore had to validate this method in this particular situation. Five percent albumin solution with ^125 ^I-albumin was sampled one minute after albumin instillation into the airspaces of the rats with ALI. This early sampling allowed to the measurement of the change in the concentration of labeled and unlabeled protein related dilution by non-labeled protein-rich alveolar edema fluid [27]. We performed the experiments in three animals and observed no change in labeled and unlabeled protein (data not shown).

### 3. *P. aeruginosa*-induced lung injury

*Pseudomonas aeruginosa *(PaO1 strain) was incubated in 125 ml of tryptic soy broth at 37°C in a rotating shaking water bath for 8 hours. The culture was then washed twice with phosphate-buffered saline, and resuspended in phosphate-buffered saline. The resulting bacterial suspension was at 1 × 10^9 ^CFU/ml. ALI was produced according to the method described by Pennington and Ehrie [28]. Under short ether anesthesia, a small midline incision was made on the neck ventral surface after swabbing it with ethanol. The trachea was exposed by blunt dissection. Using a 28-gauge needle, 0.5 ml/kg of bacterial suspension was instilled into the trachea, followed by injection of 0.5 ml of air. The animals were studied in vivo 4 hours after instillation of bacteria.

### 4. Recombinant human activated protein C

Recombinant human activated protein C (rhAPC) was generously provided by Lilly SAS (Saint-Cloud, France). Animals were anesthetized with 60 mg/kg of ketamine associated with 1 mg/kg of chlorpromazine chlorhydrate intra-muscularly. A Silastic catheter (Dow corning Corporation, Milland, MI) was inserted into the superior vena cava via the right external jugular vein. The catheter was tunneled subcutaneously to the interscapular region. Resuspended rhAPC was then continuously administered at 300 (μg/kg/h during 4 hours intravenously. This dosage regimen was chosen according to manufacturer's recommendation, taking into account the species differences in rhAPC activity.

### 5. Bronchoalveolar lavage (BAL)

Lungs from each experimental group were lavaged with a total of 20 ml in 5-ml aliquots of PBS with 3 mM EDTA. The returned fluid was pooled and centrifuged (200 *g *for 10 min), and the cellular pellet was washed two times in PBS. A cell count was performed directly with a hemocytometer, and the total number of cells was calculated. Cellular monolayers were prepared with a cytocentrifuge and stained with Wright-Giemsa stain. Cellular morphotype differential was obtained by counting 200 cells/sample and expressing each type of cell as a percentage of the total number counted.

### 6. Cytokines assays

Levels of tumor necrosis factor α (TNFα and interleukin 6 (IL-6) were determined after treatment by use of commercially immunoassay kits (ELISA) specific for rat cytokines (respectively, Quantikine Murine rat TNFα and Quantikine Murine rat IL-6, R&D Systems, Abingdon OX, UK). The reading was performed with a microplate reader Digiscan (Spectracount Packard Instrument Company; Meriden CT USA). Levels were measured in the serum and in BAL fluids of animals from each group 4 hours post-instillation of bacteria.

### 7. Coagulation

The following parameters were measured 4 hours post-instillation of bacteria in blood samples obtained through direct aortic catheterization of anesthetized rats from each group: leukocyte and platelet counts, prothrombin time, fibrinogen and thrombin-antithrombin (TAT) complex concentrations. Measurements of TAT complexes were used as markers of thrombin formation [33]. TAT complexes were also measured in BAL fluids. Platelet and leukocyte counts were obtained on EDTA-treated uncoagulated blood. For coagulation assays, blood (four parts) was collected into sodium citrate 3.8% (one part). Platelet depleted plasma was separated by centrifugation (15°C at 2500 *g *for 15 min). Prothrombin time, fibrinogen concentration (Clauss' method) were measured immediately by standard procedures. These two parameters were measured to detect the occurrence of an overt intravascular coagulation leading to a consumptive coagulopathy and fibrinogen depletion. Other assays were performed on frozen plasma or BAL fluid stored at -80°C. TAT complexes were measured on citrated samples by an enzyme-linked immunosorbent assay method (Enzygnost® TAT, Behringwerke AG, Marburg, Germany).

### 8. Experimental groups

Animals were randomized to one of four groups with a sample size of 5 animals per group in each series of experiments (in vivo permeability, alveolar liquid clearance, BAL, cytokines, histology and coagulation studies):

- Control group (**CTR**): saline (0.5 ml/kg intratracheal injection)

*- Pa *(**PNP**): *Pa *(0.5 ml/kg of 1 × 10^9 ^cfu intratracheal inoculum)

- rhAPC group (**APC**): saline (0.5 ml/kg intratracheal injection), treated (300 μg/kg/h IV rhAPC)

- rhAPC/*Pa *group (**A-PNP**): *Pa *(0.5 ml/kg of 1 × 10^9 ^cfu intratracheal inoculum), treated (300 μg/kg/h IV rhAPC).

**Figure 1:**
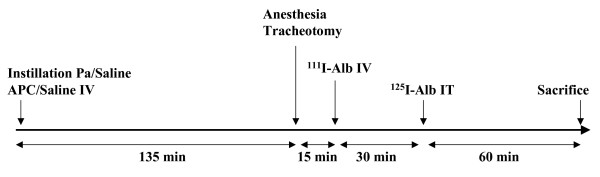
Experimental protocol over time. The animals are followed during a four hours period. After the instillation of *Pseudomonas aeruginosa *(Pa) or isotonic saline, rhAPC (APC) or saline is injected continuously during the next four hours of the experiment. After anesthesia and tracheotomy, ^111^I-labeled albumin is injected intravenously (IV), 150 minutes after the beginning of the experiment. Sixty minutes prior to sacrifice, ^125 ^I-labeled albumin is instilled intratracheally (IT). The animals are then sacrificed for each of the functionnal studies described in the method section.

For each protocol the timing was similar as represented on Figure 1

### 9. Statistical analysis

Results are presented as mean ± SEM. Data were analyzed using the Kruskall-Wallis test and the Mann Whitney test where appropriate. P values less than 0.05 were regarded as statistically significant. StatView 4.57 (Abacus Concept, Berkeley, CA, USA) software was used. A Bonfferoni correction was used for multiple analysis.

## Results

### rhAPC administration worsens oxygenation impairment in *P.aeruginosa *lung injury

Systemic arterial and airway pressures showed no significant differences between the different groups in any series of experiments (Table 1). PaO_2_/FiO_2 _ratio showed no statistically significant differences between CTR, APC and PNP groups. However, rhAPC administration significantly impaired oxygenation compared to CTR and PNP groups (Table 1).

**Table 1:** In-vivo systolic arterial pressure (SAP), airway pressure (AWP) and PaO_2_/FiO_2 _ratio after a 30 minutes stabilization period.

Group	SAP (mm Hg)	AWP (cm H_2_O)	PaO_2_/FiO_2_
CTR	127 ± 5	4.3 ± 0.4	420 ± 37
APC	118 ± 16	4.8 ± 0.6	483 ± 47
PNP	123 ± 20	4.1 ± 0.3	386 ± 33
A-PNP	104 ± 12	4.1 ± 0.6	215 ± 26^† ‡^

Data are means ± SEM. ^† ^p < 0.05 vs. CTR, ^‡ ^p < 0.05 vs. PNP.

### rhAPC does not further increase alveolar-capillary protein permeability in early *P. aeruginosa*-induced lung injury

**Figure 2:**
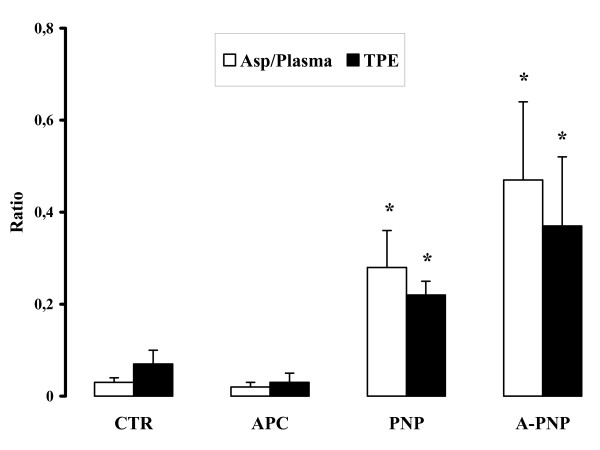
In-vivo alveolar-capillary protein permeability. Endothelial permeability, evaluated by determining leakage of the vascular marker (^111^In labeled albumin) into the alveoli as assessed by the aspirate radioactivity ratio (Asp/Plasma) (*white bars*), was significantly increased in PNP group versus control animals (0.28 ± 0.08 vs. 0.03 ± 0.01, p < 0.05). Concomitant rhAPC administration further increased this leakage compared to PNP animals. The accumulation of the vascular protein tracer into the extravascular space of the lung (TPE) (*black bars*) was increased in the PNP group versus CTR. Concomitant rhAPC administration increased TPE compared to PNP animals. Data are means ± SEM. * p < 0.05 vs. CTR.

Endothelial permeability, evaluated by the leakage of the vascular marker into the alveoli (Asp/Plasma ratio), was significantly increased in the PNP group versus control animals (0.28 ± 0.08 vs. 0.03 ± 0.01, p < 0.05). Concomitant administration of rhAPC further increased this leakage compared to the PNP group, but the difference did not reach a statistical significance (0.47 ± 0.17 vs. 0.28 ± 0.08, p = 0.2) (Figure 2). Consistent with this finding, bacterial instillation led to a significant increase in total plasma equivalents (TPE) compared to the CTR group (0.22 ± 0.03 vs. 0.07 ± 0.03, p < 0.05). rhAPC administration increased TPE in the A-PNP group compared to the PNP group, but this increase remained below a statistical significance (0.37 ± 0.15 vs. 0.22 ± 0.03, p = 0.33) (Figure 2).

### rhAPC administration induces an increase in extra-vascular lung water and a decrease in LLC

The lung wet to dry weight (W/D) ratio was significantly increased in the PNP group (4.62 ± 0.31) compared with the CTR group (3.87 ± 0.22, p < 0.05). Concomitant administration of rhAPC tended to increase the W/D ratio following *Pa*-induced ALI (5.80 ± 0.66, p = 0.07) (Table 2).

**Table 2:** Lung wet to dry weight (W/D) ratio and lung liquid clearance (LLC) for each group.

Group	W/D ratio	LLC (%)
CTR	3.87 ± 0.22	23.5 ± 2.9
APC	4.16 ± 0.34	20.3 ± 2.2
PNP	4.62 ± 0.31*	33.4 ± 2.6^†^
A-PNP	5.80 ± 0.66^†^	9.1 ± 4.3^† ‡^

Data are means ± SEM; * p < 0.05 versus CTR, ^† ^p < 0.05 vs. CTR, ^‡ ^p < 0.05 vs. PNP.

*Pa*-induced ALI was associated with a significant increase in LLC versus control animals. In contrast, rhAPC co-administration decreased significantly LLC compared with PNP animals (Table 2).

**Figure 3:**
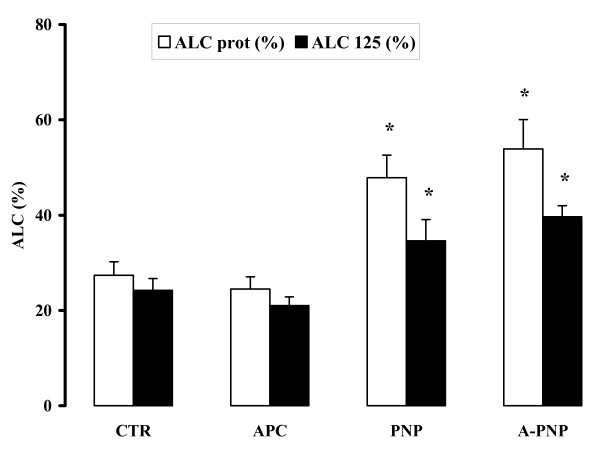
In-vivo alveolar liquid clearance (ALC). ALC was calculated from the changes in concentration, expressed in percentages, of both labeled and unlabeled protein recovered in the aspirate. ALC calculated from native protein (ALC prot) (*white bars*), showed increased values in the PNP and the A-PNP groups versus the CTR group (p < 0.05). Analysis of the results obtained from labeled proteins (ALC 125) (*black bars*) showed comparable values; both of the values in the PNP and A-PNP groups differed statistically from the CTR group (p < 0.05). Data are means ± SEM. * p < 0.05 vs. CTR.

Alveolar fluid clearance calculated from native proteins as well as labeled proteins showed increased values in the PNP group versus the CTR group (respectively, 47.9 ± 4.7 % vs. 27.4 ± 2.8 %, p < 0.05 and 34.6 ± 4.5 % vs. 24.2 ± 2.5 %, p < 0.05). Administration of rhAPC did not influence this parameter (Figure 3).

### rhAPC increases lung inflammatory cell recruitment in Pa-induced lung injury

The number of inflammatory cells recovered from the BAL fluid was significantly increased in the PNP group compared with the CTR group (9.2 ± 3.5 × 10^6 ^cells/ml vs. 1.7 ± 0.7 × 10^6 ^cells/ml, p < 0.05). This value was further increased in the A-PNP group (38.1 ± 4.4 × 10^6 ^cells/ml, p < 0.05).

More than 90% of the cells were alveolar macrophages in control and APC animals. For PNP animals, neutrophils represented 85.5 ± 4.5 % of the cells. rhAPC administration induced a significant increase of neutrophils compared to the PNP group (95.7 ± 1.3 %, p < 0.05).

### rhAPC administration induces a loss of compartmentalization of the inflammatory response

In the BAL fluid, TNFα was non detectable in CTR and APC groups. These cytokines were significantly increased in both the PNP and the A-PNP groups (p < 0.05) (Table 3). Interestingly, in the serum, TNFα was non detectable, even after *Pa *instillation. rhAPC co-administration led to a significant increase in TNFα systemic levels (p < 0.05). A comparable pattern was observed with IL-6, major levels were obtained in the A-PNP group (p < 0.05), significantly higher than in the PNP group (Table 3).

**Table 3:** TNFα, and IL-6 levels in blood serum and BAL fluid for each group.

Group	TNFα (pg/ml)		IL-6 (pg/ml)	
	Blood serum	BAL fluid	Blood serum	BAL fluid
CTR	n.d.	n.d.	171.6 ± 17.7	83.9 ± 2.1
APC	n.d.	n.d.	142.9 ± 16.9^†^	90.4 ± 14.5
PNP	n.d.	957.2 ± 337.2^†^°	225.4 ± 57.0^†^	2706.0 ± 1720.1^†^°
A-PNP	19.3 ± 14.1^†^° ^‡^	1697.0 ± 180.8^†^° ^‡^	470.7 ± 111.5^†^° ^‡^	4452.4 ± 1283.4^†^°

n.d.: non detectable.

Data are means ± SEM.^† ^p < 0.05 vs. CTR, °p < 0.05 vs.APC, ^‡ ^p < 0.05 vs. PNP.

### The levels of alveolar coagulation biomarkers are majored by rhAPC administration in *P.aeruginosa*-induced lung injury

A significant decrease in white blood cell count was observed in *Pa *inoculated groups compared with the control group (p < 0.05). A decreased platelet count was observed in the APC group (Table 4). Concerning plasma fibrinogen levels, a significant increase was observed in the PNP group compared with control animals (p < 0.05), whereas a significant decrease was observed in the APC and the A-PNP groups compared with controls (p < 0.05) (Table 4). Plasma TAT complexes were comparable between all the groups.

**Table 4:** Standard coagulation parameters for each group at 4 hours

Group	WBC (10^9^/l)	Plt (10^9^/l)	PT (%)	Fibrinogen (g/l)
CTR	6.69 ± 0.76	793 ± 59	52 ± 3	2.38 ± 0.13
APC	5.10 ± 0.91	580 ± 53^†^	43 ± 2	1.93 ± 0.11
PNP	3.63 ± 0.65^†^	850 ± 14°	52 ± 1	3.22 ± 0.36^†^°
A-PNP	2.77 ± 0.45^†^	778 ± 24°	46 ± 3	2.36 ± 0.11^‡^

WBC: white blood cells, Plt : Platelets, PT : Prothrombin time

Data are means ± SEM.^† ^p < 0.05 vs. CTR, ° p < 0.05 vs. APC, ^‡ ^p < 0.05 vs. PNP.

**Figure 4:**
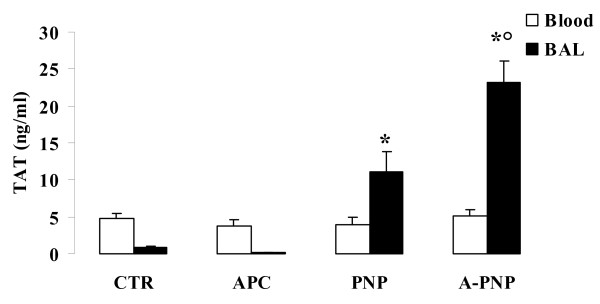
Serum and bronchoalveolar lavage (BAL) fluid Thrombin-Antithrombin complexes (TAT). There was no significant variation between groups in blood serum TAT complexes (*white bars*). However, there was a significantly increase in BAL fluid TAT complexes (*black bars*) in the PNP group compared to the CTR group. rhAPC administration in *Pa*-induced lung injury animals further increased BAL fluid TAT complexes. Data are means ± SEM. * p < 0.05 vs. CTR and APC, ° p < 0.05 vs. PNP.

In the alveoli, analysis of the BAL fluid showed a statistically significant increase in TAT complexes in the PNP group versus CTR group (11.06 ± 2.76 vs. 0.92 ± 0.18 ng/ml, p < 0.05). Moreover, TAT complexes in the BAL fluid were significantly increased in the A-PNP group, compared with the PNP group (23.17 ± 2.89 vs 11.06 ± 2.76 ng/ml, p < 0.05) (Figure 4).

## Discussion

In our study, *Pa*-induced lung injury led to an increase in alveolar-capillary barrier permeability and rhAPC administration tended to increase extra-vascular lung water in the A-PNP group. Analysis of lung fluid movement showed an increase in alveolar lung clearance in pneumonic animals. If the epithelial transport was relatively preserved in our model, endothelial function alterations were observed with a decrease of lung liquid clearance after rhAPC administration in pneumonic animals. Consistent with these findings, rhAPC treated animals had a loss of lung cytokines compartmentalization as assessed with TNFα and IL-6 systemic levels. The analysis of coagulation parameters showed a major increase of TAT complexes in the BAL fluid associated with the increased permeability.

In the first part of our study, we showed an increase in the alveolar-capillary barrier permeability after instillation of the bacteria. Our results are consistent with previous findings, we showed a leak of the vascular tracer into the alveoli (Asp/Plasma ratio) [7,27] associated with an increase in the total plasma equivalents (TPE) [26].

Concomitant administration of rhAPC in *Pa*-induced lung injury group further tended to increase extra-vascular lung water in the A-PNP group. Murakami et al. showed that APC pretreatment could reduce permeability disorders and histological damages in a model of LPS-induced pulmonary vascular injury [29]. These effects were related to APC ability to inhibit pulmonary accumulation of leukocytes. Our results are not consistent with these findings; however, the type of injury induced in our model differs from that of Murakami et al. First, in our model, the lung injury was induced by a living bacteria instillation and not only by a component of this bacteria; toxic substances (such as exotoxins) released by living *P. aeruginosa *could explain the severity of the injury observed in our study. Second, in the study of Murakami et al., the injury only involved the endothelial side of the alveolar-capillary barrier, when our model involves both sides [3]. Finally, in our model, rhAPC administration was concomitant with the *Pa*-induced lung injury; animals were pre-treated in the study of Murakami et al.

An important and unexpected finding of this study was that rhAPC reduced LLC in the A-PNP group. LLC, *i.e. *the portion of the instillate which left not only the alveoli but also the lung, was increased by the bacterial insult in the PNP group. Concomitant rhAPC administration was responsible for a significant alteration of this parameter. Pulmonary edema resorption involves two main pathways: vascular and lymphatic [30]. The alveolar-capillary barrier permeability was altered in both the A-PNP and the PNP groups. The lung W/D weight ratio is the result of the combination of ALC and LLC. In the PNP group, the balance between alveolar liquid clearance and lung liquid clearance could limit the increase in the lung W/D weight ratio and therefore preserve gas exchange. In the A-PNP group, the alteration of the endothelial barrier was probably responsible for the decrease in the lung liquid clearance, as a consequence, lung W/D weight ratio increased and gas exchange was altered. In this hypothesis, the worsening of the oxygenation was not related to alveolar flooding but to mismatched of V/Q. Our findings are consistent with previous results of others indicating that alveolar epithelium is more resistant than the lung endothelium to acute lung injury [31-33]. We have also previously shown in another model that this discrepancy between ALC and LLC could be related to the endothelial barrier [34]. Here, the increase in endothelial permeability in the A-PNP group seems to overwhelm alveolar transport and as a consequence be responsible for alveolar flooding.

We found a preservation of alveolar liquid clearance (ALC) assessed by native and labeled albumin concentration variations. Four hours after bacterial instillation, ALC was significantly increased compared to the control group. We observed an increase from 27% to 48%, consistent with previously published studies which showed an increase from 30% to 45% in a comparable model [27]. In these studies, the increase in ALC was related to a TNFα-dependent mechanism [27] since it was completely prevented by anti-TNFα antibody pretreatment and reproduced by TNFα instillation alone. In our study, we found a high BAL fluid TNFα level in *Pa*-induced lung injury groups, which can explain, at least partially, ALC increase.

A large number of studies has demonstrated the relationship between systemic inflammation and the coagulation system [35]. With inflammation, many cytokines are produced, among them TNF has been shown to inactivates natural inhibitors of coagulation and attenuates fibrinolysis, and IL-6 to activate coagulation [36]. We observed a major increase in BAL fluid pro-inflammatory cytokines levels in the PNP group compared to control animals, whereas these cytokines were non detectable or only slightly increased in the blood. TNFα and IL-6 are mainly released from macrophages and neutrophils both activated by different products from *P.aeruginosa *[37]. In the PNP group, locally released cytokines remained within the alveolar space [38], and blood serum cytokines levels were lower than in the alveolar compartment. In the A-PNP group, we observed a significant increase in both blood serum TNFα and IL-6 levels compared to CTR and PNP groups standing for a loss of compartmentalization of the inflammatory response associated with systemic mediators release [38,39].

rhAPC administration in *Pa*-induced lung injury led to a 4-fold increase in TAT complexes in the BAL fluid, whereas blood serum values were not significantly different between groups. Activated PC is a strong inhibitor of factors Va and VIIIa and downregulates thrombin formation. In our model of experimental lung injury, the increased TAT levels in the BAL fluid could represent an indirect witness of the APC induced-increase in capillary-alveolar permeability, leading to the migration and accumulation of TAT complexes at the alveolar side. The lack of a concomitant increase in plasma TAT levels is poorly consistent with this mechanism. It is also well documented that alveolar epithelial cells are able to generate a substantial amount of thrombin independently of any intravascular coagulation activation. Alveolar macrophages are able to express tissue factor-dependent pro-coagulant activity and synthesize factor V that accelerates thrombin formation via the prothrombinase complex [40]. The rat fetal distal lung epithelium has been used in vitro to study epithelial thrombin formation and it has been shown, by Chan et al, that lung epithelial cells promote thrombin generation via the expression of factor VII dependent tissue factor activity [41]. On the other side, these epithelial cells have also significant anticoagulant properties. Bacterial LPS increases the expression of thrombomodulin in alveolar macrophages and this effect could enhance the activation of Protein C in the thrombin-thrombomodulin complex. Epithelial cells express on their cell surface glycoaminoglycans able to bind to plasma Antithrombin (AT) and leading to the generation of TAT complexes in the alveolar fluid [41]. We could document in a previous study that the levels of alveolar TAT complexes were greatly enhanced when recombinant human AT was infused intravenously in the same *Pa *model, showing that a substantial amount of thrombin had been generated in the alveolar fluid [42]. Therefore, the possibility must be considered that the increase in TAT alveolar levels observed in the present study after intravenous APC infusion, might reflect the perturbation of the complex pro and anti coagulation balance at the alveolar level. Additionally, the effects of rhAPC on endothelial permeability and epithelial cells are not uniform. Experimental studies have shown that they may differ according to the animal model, the dosage regimen, and the infusion route. For instance, Zeng and al. have shown that contrasting with a lack of effect at low dose, higher dose (≥ 1 μg/mL) of rhAPC could induce an early endothelial cell leakage [43]. These data suggest that interpretation of the published in vitro and in vivo data of rhAPC and endothelial permeability should take into consideration the concentrations of rhAPC used or achieved. In the present study we used the dosage of rhAPC recommended by the manufacturer, taking into account that species differences in APC activity require higher dose in the rat model. Nevertheless we consider that our results support the possibility that the infusion of rhAPC at the early stage of bacterial pneumonia might disturb the complex coagulation balance at the alveolar level and impede the initial favorable effect of coagulation activation.

Indeed, during the earliest phase of acute lung injury, the epithelial as well as the endothelial side of the alveolar-capillary barrier are involved with a fibrin deposition on both sides reflecting the shift in the alveolar coagulation/fibrinolysis balance [10]. During the first 72 hours of human ARDS, the procoagulant activity is maximal and fibrinolytic activity is decreased; then, from the fourth to the fifteenth day, fibrinolysis inhibition persists whereas the procoagulant activity is moderately increased [12,15,44,45]. In such a situation, the inhibition of fibrin formation appears to be an attractive therapeutic approach. However, the possibility of deleterious effects of enhancing fibrinolysis or inhibiting intra-alveolar coagulation in the early stages of ARDS has to be considered. The early inhibition of thrombin formation by activated PC at the endothelial side might impede the natural plugging of the capillary alveolar leak induced by the bacterial insult. This possibility is further supported by recent results from our laboratory showing in the same rat model that inhibition of thrombin formation by recombinant AT also increases lung permeability and aggravates lung damage [42]. Consistent with this hypothesis, rhAPC administration in *Pa*-induced lung injury tends to increase extra-vascular lung water with a loss of compartmentalization of the inflammatory response. This finding underlines the importance of early fibrin formation to limit the extent of injury.

This study has several limitations before the extrapolation of the results to clinical situations. Concerning the design of the study, we used a model of acute lung injury in the rat at the very early phase of the injury; patients are usually seen at a later phase. We used recombinant human activated C protein in a rat model, the species difference can explain the higher dose used in our study, however, the dose was chosen according to the manufacturer's recommendations at the time of the study. Finally, APC was administered concomitantly, which is better than in pre-treatment, but still far from the clinical use.

On the results obtained, we only have a limited number of animals in each group and the measure of the alveolar liquid clearance is always difficult with an increase of the alveolar barrier permeability, however, we used the classical corrections to obtain a reliable parameter [27]. Finally, the measure of the endothelial and epithelial permeability relies on the study of a double flux of markers. The results obtained reflect a combination of these two barriers permeabilities, a fine distinction could only be studied through morphological data.

## Conclusion

Our results show that administration of 300 μg/kg/h of rhAPC in the early stage of *Pa*-induced lung injury tends to increase lung edema formation with a loss of the inflammatory response compartmentalization. These effects could be related to the inhibition of thrombin formation by rhAPC, suggesting a probable beneficial role of early coagulation activation in ALI limiting both extents of injury and permeability disorders. In a later stage of ALI, the modulation of haemostasis by rhAPC could be of value to restore the fibrinolytic capacities and limit the intensity of inflammatory reaction [46].

## Authors' contributions

LR and FC were responsible for the acquisition of the data. AT performed all the measurements related to the coagulation system. TP performed the radioactive labelling of the albumin (I^131^). RN made substantial contributions to the drafting of the manuscript and the analysis of the data. FF and BG were involved in the acquisition of the data, the design and the conception of the study as well as the drafting of the article. All the authors read and approved the final manuscript.
